# 2016. CLABSIs in the Time of COVID

**DOI:** 10.1093/ofid/ofac492.1640

**Published:** 2022-12-15

**Authors:** Maria Rosa Velasquez Espiritu, Mary Dundas, Donald S Chen, Marina Keller

**Affiliations:** New York Medical College - Metropolitan Hospital / Westchester Medical Center, New York, New York; Westchester Medical Center, Valhalla, New York; Westchester Medical Center, Valhalla, New York; Westchester Medical Center, Valhalla, New York

## Abstract

**Background:**

With the COVID-19 pandemic, acute care facilities experienced an initial increase in hospital-acquired infections, most notably Central Line-Associated Bloodstream Infections (CLABSIs). Interestingly, the positive correlation between COVID-19 and CLABSIs appeared to wane post surge. We sought to define the pre and post-pandemic CLABSI rates.

**Methods:**

Single-center, retrospective review of the CLABSIs reported to the National Healthcare Safety Network (NHSN) for the five years previous to COVID and through to the First Quarter of 2022. Our center is a quaternary care, level 1 trauma center in New York, which serves a highly ill critical care population, including solid organ and bone marrow transplants, acute leukemias, and patients requiring extracorporeal membrane oxygenation (ECMO).

**Results:**

Between January 2015 and December 2019, our Medical Intensive Care Unit (MICU) reported 15 CLABSIs with an overall Standardized Infection Ratio (SIR) of 1.007. During the second winter surge of COVID, we saw a sharp rise in CLABSIs. Between October 2020 and March 2021, our MICU reported eight CLABSIs, with an overall SIR of 4.601 and a p-value of 0.0005 (CI 2.137 – 8.737), representing a statistically significant increase (p-value 0.0019) in comparison to prior years. Of these patients, seven had COVID. All patients received high-dose steroids. The average number of days between hospital admission to CLABSI was 16.38 days, and mortality was 87.5%. Since that spike in CLABSI, we have reported seven CLABSIs in NHSN for a SIR of 2.026. After 2019 patients with CLABSI tended to be younger and had a higher Body Mass Index.

**Figure:**
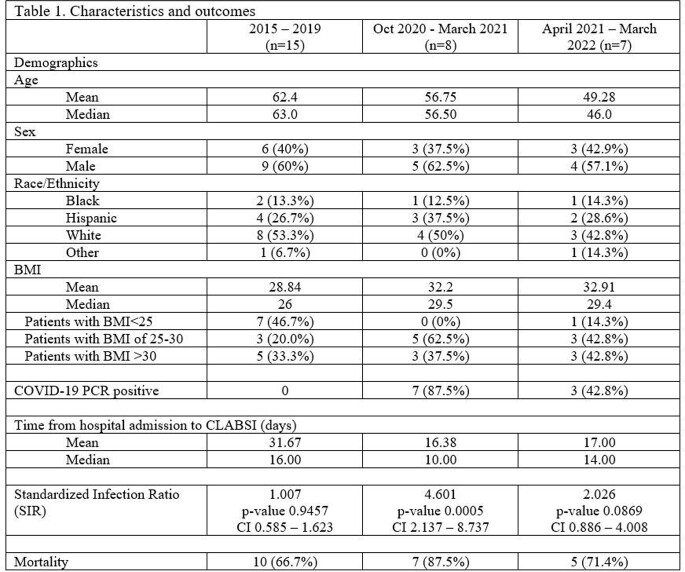

**Conclusion:**

We reported a spike in CLABSIs in our adult medical Intensive Care during the second wave of COVID-19 that affected the Northeast United States in the winter of 2020/2021. Despite experiencing a sharp rise, our CLABSI rates are returning to pre-pandemic low levels even during subsequent surges in COVID-19, including the recent Omicron surge. It remains to be determined if the improvements in infection control measures, differences in the patient illness severity, and/or variations of COVID management have contributed to the stabilization of the CLABSI rate.

**Disclosures:**

**All Authors**: No reported disclosures.

